# County Councils and Public Health

**Published:** 1916-09-16

**Authors:** 


### The Hospital

The Workers' Newspaper of Administrative Medicine and Institutional Life, Administration, National Insurance and Health.

No. 1580, Vol. LX. SATURDAY, SEPTEMBER 16, 1916. PRICE ONE PENNY.

### COUNTY COUNCILS AND PUBLIC HEALTH.

For the most part local government has been marking time since the outbreak of the war. With the absorption of the energies of members and officials in various kinds of war work, with the depletion of staffs by the demands of military service, and with the stern call to financial retrenchment it was inevitable that the activity of local authorities for the social welfare should sustain a serious set-back. Nor is it merely marking time which is to be regretted; it is to be feared that in the name of economy there has been in some departments of local government, notably education, a retrogression which was probably not inevitable and will certainly prove costly. In these circumstances it is the more gratifying to notice one sphere in our local administrative system in which during the war some progress has actually been achieved, small though it may be, but giving promise of greater things under the more normal conditions that we hope peace will restore to us.

Up to the present the County Councils have had but little part in the administration of the law relating to public health. (In thus speaking of County Councils we leave out of consideration, of course, the London County Council, with its much wider powers, whose creation under this title was a practical jest on the part of the late Lord Ritchie.) But in 1915 as the result of a small and unobtrusive Act of Parliament the County Councils, in principle at least, were given a new *rôle* in relation to public health. Some of our Acts of Parliament mean much less than their titles imply: others mean much more. The Notification of Births (Extension) Act, 1915, is in the latter category. The nominal purpose of this statute was to put into force the Notification of Births Act, 1907, in all parts of the country where it had not been adopted by the local authorities. But, incidentally, it gave to County Councils all the powers of the Public Health Acts in respect to the care of expectant mothers, nursing mothers, and young children. They were enabled to appoint health visitors, provide skilled attention for the confinement of poor women, and also establish centres for the treatment of both mothers and children. These powers, tacked on to the advisory and supervisory functions already exercised by County Councils in regard to other health matters, should enable them to make a most valuable contribution towards the great task of preserving and strengthening infant life, now made all the more important by the waste of manhood in war. They will be able to fill in the gaps not otherwise provided for by local organisation; they can prevent overlapping and promote co-ordination. The large cities and the county boroughs generally wherein the County Councils have no jurisdiction are, of course, quite capable of working out their own salvation in these as in other matters pertaining to public health. But in the case of some of the subordinate local authorities the largeness of their area and the smallness of their population render the efficient administration of all the provisions of public health legislation practically impossible. They cannot employ a health visitor, for instance, for her whole time, and the salary they can afford to offer will not secure a woman with the best qualifications for the work, whilst her travelling expenses will be a heavy item relative to the service she can render. It will obviously be a better course for such authorities to hand over the whole business to their County Council. The County Council can map out the area in question into compact areas, not according to the local government boundaries but according to travelling facilities. It can combine in one officer for each area the duties of health visitor, school nurse, and inspector of midwives. In this way time and expense in travelling will be saved and the services of better qualified women obtained. Moreover, by this plan will be avoided to some extent the repetition of visits by different officials, which is not unnaturally resented by the poor. It is true that a County Council is not a sanitary authority and that its health visitors will have to report any sanitary defects in the houses visited to an authority other than their own employers, but with good feeling on both sides this should not prove a drawback of any practical importance. On the whole, therefore, it may be expected that by the exercise of their new powers the County Councils, as regards health visitors, may enable the smaller districts to reach in time the standard obtained by some of the larger municipalities, which have provided one health visitor for every 500 births per annum. By the end of last year—the enabling Act having only come into force on September 1, 1915—the County Councils had already appointed 185 health visitors, ten of whom gave the whole of their time to the office, the remainder sharing it with other duties. These figures, it must be admitted, represent a very fair beginning.

With the development of the work there seems no good reason why County Councils should not, by friendly arrangement with the local authorities, arrange for the establishment of maternity centres in places where they would be of service to a population scattered over several local government areas. In regard to the provision of midwives, too, the new powers of the County Council should prove particularly useful. In towns and even in villages of any size competent midwives are usually obtainable on terms within the means of all but the very poorest. But in remote, sparsely populated country districts a resident midwife could not obtain a livelihood, and to engage one from the nearest town is too costly for many poor women. In such circumstances the County Council, through its health visitor, may under the Act of 1915 provide the services of a trained midwife in place of such haphazard and untrustworthy attention as poor women could otherwise secure. It is a little thing, but it has its human importance, and it is in looking after these little things of human importance that our local government system serves the community equally with its large schemes of social amelioration.

